# Hybrid procedure using perineal and abdominal approaches for radical prostatocystectomy: initial experience with 16 select cases

**DOI:** 10.1186/2193-1801-2-348

**Published:** 2013-07-29

**Authors:** Yozo Mitsui, Hiroaki Yasumoto, Haruki Anjiki, Chiaki Koike, Naoko Arichi, Takeo Hiraoka, Masahiro Sumura, Satoshi Honda, Mikio Igawa, Hiroaki Shiina

**Affiliations:** Department of Urology, Shimane University School of Medicine, 89-1 Enya-cho, 693-8501 Izumo, Japan

**Keywords:** Bladder cancer, Radical cystectomy, Urethrectomy, Prepubic, Perineal

## Abstract

**Objectives:**

To validate the feasibility and implications of a hybrid procedure using perineal and abdominal approaches for a radical prostatocystectomy.

**Methods:**

Between March 2007 and May 2012, we performed 16 prostatocystectomy and simultaneous urethrectomy under a hybrid procedure using perineal and abdominal approach for advanced bladder cancer. The hybrid procedure was selected in each case, because of prostatic urethra involvement in 13 and prior treatment in 3 (irradiation, radical retropubic prostatectomy, and sigmoidectomy, respectively). Two surgical teams, one responsible for the perineal approach and the other for the abdominal portion, performed the operation.

**Results:**

The median operation time for the prostatocystectomy procedure was 207 minutes and median intraoperative blood loss was 1665 ml. The en bloc removal of the specimen was perfectly performed and no intraoperative difficulties and intraoperative complications such as rectal injury were recognized in all cases. As for postoperative complications associated with the exaggerated lithotomy position, neurologic complications and rhabdomyolysis which could be treated conservately were found in 1 case. Although 5 patients died from distant metastasis, local recurrence was not seen in any of the 16 patients during the follow-up period.

**Conclusion:**

The hybrid procedure using perineal and abdominal approach for radical prostatocystectomy is a well-organized procedure that can provide good visualization of the surgical structure around the prostate, leading to a reduction in or prevention of local recurrence and surgical complications even in the selected patient.

## Introduction

A radical cystectomy with pelvic lymph node dissection remains the mainstay treatment for patients with muscle-invasive bladder cancer as well as those with non-invasive tumors who fail intravesical therapy (Madersbacher et al. 2003; Ghoneim et al. 2008; Yafi et al. 2010). Prior pelvic medical or surgical treatment makes a radical cystectomy procedure difficult, because it often causes deep adhesions with surrounding tissues. Rectal injury is one of the most important complications during pelvic surgery and can lead to wound sepsis, pelvis abscess, rectourethral fistula, and even death, with a previous transurethral procedure and pelvic radiotherapy thought to be risk factors (Kheterpal et al. 2010). Hence, several technical modifications are required to reduce intraoperative complications in these cases.

A simultaneous urethrectomy and total prostatocystectomy procedure is indicated in male patients with urethral involvement by cancer or when there is a risk of urethral re-involvement, such as multifocal urothelial carcinoma, a positive prostatic urethral margin, carcinoma in-situ (CIS), and prostatic stromal invasion (Cho et al. 2009; Van Poppel 2006). However, such tumors are generally aggressive in nature and most patients who experience recurrence after radical cystectomy die within 15 months (Mitra et al. 2011). The incidence of local recurrence after a radical cystectomy varies from 6% to 18%, and patients presented with both local and distant recurrence have a poor prognosis (Yafi et al. 2010; Mitra et al. 2011; Hassan et al. 2006). In addition to the vascular and/or lymphatic route and surgical margin status, seeding and implantation of tumor cells may also cause local recurrence after a radical cystectomy (Herawi et al. 2006; Guven et al. 2007). Thus, en bloc removal of the specimen is required for successful radical oncologic surgery.

A hybrid procedure using both perineal and abdominal approaches for a prostatocystectomy was regularly performed prior to the 1950s, while that is now rarely chosen (Gibod & Steg 1979). However, this procedure has several advantages, including (1) better exposure of the urethra, prostate-seminal pedicles, and puboprostatic ligaments; (2) a total urethrectomy can be concurrently performed; (3) continuity of the main specimen is preserved; and (4) accidental perforation of the rectum can be avoided, even in patients with prior radiation therapy (Gibod & Steg 1979; Kishev 1978; Crawford & Skinner 1980; Nishiya & Crawford 1997). In consideration of these advantages, a hybrid procedure using perineal and abdominal approach could be advocated for cases with a history of pelvic irradiation or operation, or those indicated for a urethrectomy.

We have performed perineal radical prostatectomy (PRP) procedures for treating localized prostate cancer since December 2000 and developed a novel extended method (ePRP), which enables removal of the entire prostate en bloc together with the dorsal vein complex (DVC) and lateral pelvic fascia (Inoue et al. 2010). Our ePRP method provides better cancer control and a shorter operation time than with a conventional PRP procedure. In addition, we speculated that this technique could be applied to establish an ideal hybrid procedure using both perineal and abdominal approaches during a prostatocystectomy procedure. Here, we present results from a small series of advanced bladder cancer patients who underwent our hybrid procedure for a prostatocystectomy.

## Materials and methods

### Patients and follow-up

Between March 2007 and May 2012, 16 patients with advanced bladder cancer underwent a prostatocystectomy with our hybrid procedure that employs perineal and abdominal approaches at our institution. Our indications for this hybrid procedure include (i) previous pelvic operation or irradiation with resultant dense adhesions and scarring, and (ii) indication for a urethrectomy because of the possibility of carcinomatous involvement of the prostatic urethra with extension into the prostatic stroma or CIS. Preoperative staging parameters included computed tomography, magnetic resonance imaging of the pelvis, bone scan, cystourethroscope, transurethral tumor biopsy, and 3- and 9-hour lateral verumontanum biopsy findings. All 16 patients underwent pelvic lymph node dissection during the operation. Surgical duration was measured from the time of perineal skin incision until harvesting the prostatocystectomy specimen and starting the diversion. Routine postoperative follow-up examinations were conducted at 3-month intervals from years 1 to 5, then annually thereafter. Computed tomography, bone scan, and chest radiography examinations were scheduled for 4 months after surgery in years 1 to 2 and annually thereafter, unless otherwise clinically indicated. Local recurrence was defined as that occurring within the soft tissue field of exenteration, while distant recurrence was that occurring outside the pelvis.

### Surgical technique

**Figure 1:**
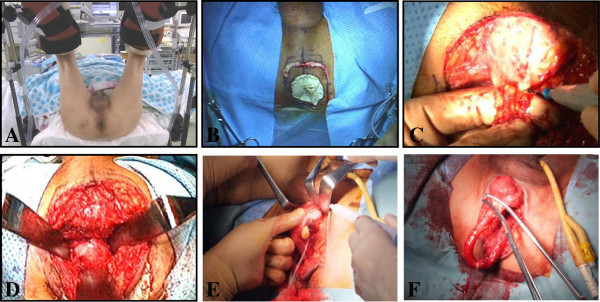
**Perineal surgery under an exaggerated lithotomy position. A**. Following general anesthesia induction, the patient is placed in an exaggerated dorsal lithotomy position. **B**. An inverse U-shaped incision is made on the apex of the middle perineum between the bilateral ischial tuberosities. **C**. The central tendon is incised with cauterization. **D**. Denonvilliers’ fascia is identified after blunt division of the rectourethralis muscle. **E**. The corpus spongiosum is sharply dissected from the corpora cavenosa, with the dissection continued distant until the fossa navicularis and external urethral meatus are reached. **F**. The urethra is incised at the level of the urethral fossa navicularis. correct virsion

Two surgical teams, one responsible for the perineal approach and the other for the abdominal portion, participated in the operation. The perineal approach was performed by the same surgeon (I.M.) in all cases. Each operation was performed as follows. After general anesthesia with an epidural catheter for postoperative pain control, the patient is placed in an exaggerated dorsal lithotomy position (Figure 1A). The “perineal” surgeon starts first. An inverse U-shaped incision is made on the apex of the middle perineum between the bilateral ischial tuberosities (Figure 1B), then the ischiorectal fossa on each side of the rectum is bluntly developed and the central tendon incised with cauterization (Figure 1C). Denonvilliers’ fascia is identified after blunt division of the rectourethralis muscle (Figure 1D). Next, an 18 F Foley catheter is placed and the corpus spongiosum is sharply dissected off the corpora cavenosa after incision of Colles’ fascia and Buck’s fascia, and the dissection is continued distantly until the fossa navicularis and external urethral meatus are reached (Figure 1E). Using a catheter, the urethra is clamped at the level of the urethral fossa navicularis and incised (Figure 1F). After evulsion of the catheter, the clamp is replaced with a 3–0 absorbable suture ligature.

**Figure 2:**
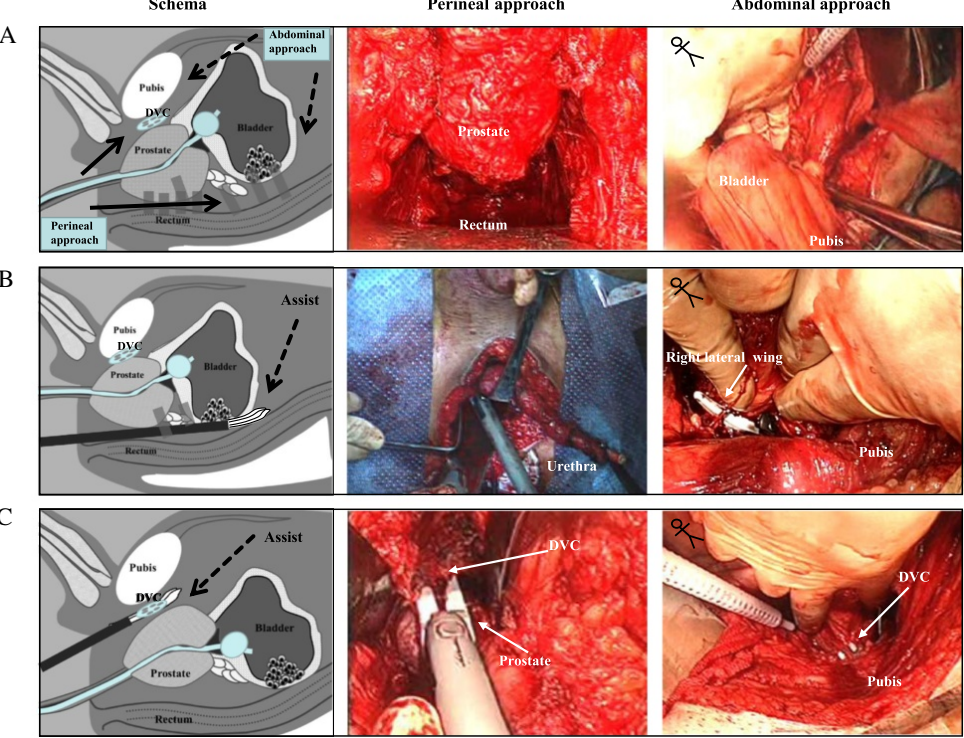
**Simultaneous perineal and abdominal surgery under a lithotomy position. A**. After mobilizing the peritoneum from the Retzius space to the iliac bifurcation on the abdominal side, the perineal team dissects between the layers of Denonvilliers’ fascia behind the prostate and seminal vesicles. **B**. Division of the bilateral pedicles is performed with the aid of both the perineal and abdominal teams. **C**. The puboprostatic ligaments and dorsal vein complex are safely divided using a sealing surgical device through a perineal approach.

Next, the patient is moved to a lithotomy position and the “abdominal” surgeon proceeds. At this stage, both “perineal” and “abdominal” operations are simultaneously performed by each team. On the “abdominal” side, a midline lower abdominal incision is made between the umbilicus and symphysis pubis. The peritoneum is mobilized from the Retzius space up to the iliac bifurcation (Figure 2A). On the “perineal” side, the posterior layer of Denonvilliers’ fascia is incised at the base of the prostate and the posterior aspects of the seminal vesicle are developed. Both ampullae of the vas are divided and the seminal vesicles are dissected. Blunt dissection is used to develop the rectovesical cul-de-sac (Figure 2A). When this blunt dissection reaches the posterior peritoneum, that is incised just above its reflection over the rectum from the abdominal side. Then, the bilateral endopelvic fascias are incised and the incisions extended, and both lateral pedicles containing the neurovascular bundles are divided with a sealing surgical device through the “perineal” approach.

**Figure 3:**
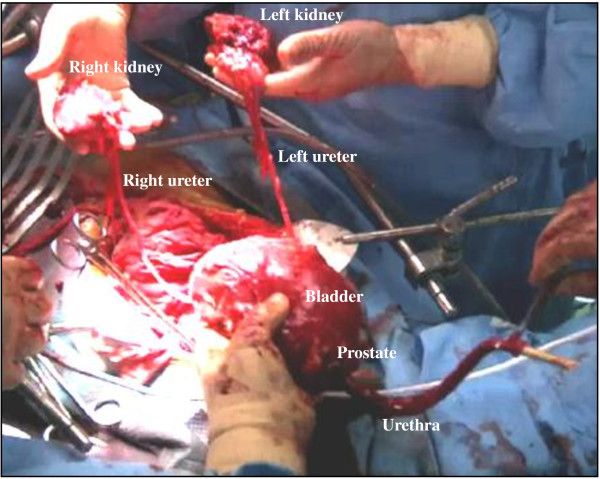
**The specimen is removed en bloc and continuity of the main portion is preserved.** The specimen shown here is from case 12, who simultaneously underwent a bilateral total nephroureterectomy.

Division of the bilateral posterior and lateral pedicles is performed with a sealing surgical device through the “perineal” approach in sequence (Figure 2B). These processes can be also observed from the “abdominal” side and the “abdominal” team assists the “perineal” operation (Figure 2B). Thereafter, the bilateral puboprostatic ligaments and DVC are safely divided with a sealing surgical device, and the urethra is pulled into the small pelvis (Figure 2C). Using this technique, the DVC can be adequately controlled (Inoue et al. 2010). After excision of the bilateral ureters, en bloc removal of the specimen can be completed (Figure 3). The levator ani and subcutaneous tissue are approximated in the middle perineum, with a closed-type drain placed on the rectum and bladder.

## Results

**Figure 4:**
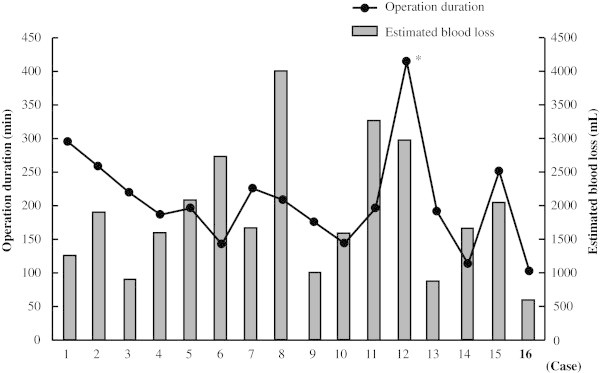
**Operation duration and estimated blood loss in 16 patients.** Operation duration tended to follow a learning curve, except for case 12, who simultaneously underwent a bilateral total nephroureterectomy. On the other hand, estimated blood loss did not follow a learning curve.

Background information, clinical characteristics, and pathological data for the 16 patients are shown in Table 1. Median patient age at surgery was 66 years old (range 55–77 years) and the median follow-up period for patients alive was 20 months (7–29). The hybrid procedure was selected in each case because of the possibility of prostatic urethra involvement in 13 and prior treatment in 3 (pelvic irradiation, radical retropubic prostatectomy, sigmoidectomy). Fifteen patients (93.7%) had an ilial conduit urinary diversion, while the remaining patient (case 12) (1/16, 6.3%) did not received urinary diversion because of hemodialysis and underwent a bilateral total nephroureterectomy for renal tumors during the same operation. The tumors in 8 (50.0%) patients were pathological grade 2 and the remaining 8 (50%) were grade 3. Pathological stage was pTis in 4 cases, pT1 in 2 cases, pT3a in 2 cases, pT3b in 2 cases, and pT4a in 6 cases. Six (37.5) patients had lymph node metastasis and all 16 had negative surgical margins. The median operation duration and estimated blood loss were 196 minutes (102–415) and 1665 ml (600–4000), respectively. As shown in Figure 4, the operation duration tended to follow a learning curve, except for case 12. On the other hand, estimated blood loss did not follow a learning curve.

**Table 1:** **Clinical characteristics and pathological data of 16 patients**

Median (range):	
Age, years	66 (55–77)
Follow-up, months	15 (2–29)
N (%):	
Indication for abdominoperineal approach	
Possibility of prostatic urethra involvement	13 (81.1)
History of irradiation	1 (6.3)
After radical retropubic prostatectomy	1 (6.3)
After sigmoidectomy	1 (6.3)
Urinary diversion	
Ilieal conduit	15 (93.7)
Not performed	1 (6.3)
Pathological tumor grade	
2	8 (50.0)
3	8 (50.0)
Pathological stage	
pTis	4 (25.0)
pT1	2 (12.5)
pT3a	2 (12.5)
pT3b	2 (12.5)
pT4a	6 (37.5)
Pathological node status	
pN0	10 (62.5)
pN+	6 (37.5)
Surgical margins	
Negative	16 (100)
Positive	0
Median (range):	
Operation duration, min	196 (102–415)
Estimated blood loss, mL	1665 (600–4000)
N (%:)	
Complication:	
Neurapraxia and rhabdomyolysis	1 (6.3)
Ileus	1 (6.3)
Adjuvant chemotherapy	
Not administered	5 (31.3)
Administered	11 (68.7)
Site of occurence	
Local	0
Distant	5 (31.3)

En bloc removal of the specimen was completed in all cases, and no intraoperative difficulties or complications such as rectal injury occurred. Even in the 3 cases with prior medical or surgical treatment, the operation was safely completed. As for postoperative complications, ileus was found in 1 patient (case 12) who had a history of ileus after a sigmoidectomy. In addition, neurapraxia and rhabdomyolysis associated with the exaggerated lithotomy position were found in 1 (case 15), who was treated in a conservative manner. A total of 11 patients (68.7%) received adjuvant chemotherapy. Although 5 patients died from distant metastasis, local recurrence was not seen in any of the 16 patients during the follow-up period.

### Comments

The current standard treatment for muscle-invasive bladder cancer is a radical cystectomy with an extended bilateral pelvic lymph node dissection (Madersbacher et al. 2003; Ghoneim et al. 2008; Yafi et al. 2010). Most urologists likely select a retropubic approach for radical surgery of the bladder, even in patients who have received prior pelvic irradiation or surgical treatment. However, pelvic adhesions often occur after those treatments, and can obscure and obliterate the anatomic planes, thus rendering a retropubic prostatocystectomy after these treatments as a challenging procedure. In the present series, we performed a prostatocystectomy under a hybrid procedure using perineal and abdominal approaches for 3 patients who receive previous treatments, including pelvic irradiation in 1, a radical retropubic prostatectomy in 1, and a sigmoidectomy in 1, with all of the present procedures safely and readily completed. Furthermore, accidental perforation of the rectum was easily avoidable, because our technique offers an excellent view of the blunt dissection of the rectum, making it easier to take down the rectum from the prostate and mobilize the distal third of the bladder (Kishev 1978).

While distant recurrence may reflect micrometastatic disease at the time of surgery, local recurrence invariably signifies inadequate surgical resection. In addition, seeding and implantation of cancer cells may be an integral part of bladder cancer surgery in terms of local recurrence (Herawi et al. 2006; Guven et al. 2007). The present hybrid procedure facilitates dissection of the urethra, preserves continuity of the main specimen, and contributes to complete resection of the tumor. In this series, no patient had a positive surgical margin, even in advanced stage cases (T3 or T4). Furthermore, local recurrence was not seen in any of the 16 patients during the follow-up period, though 5 died from distant metastasis. Thus, our findings indicate an acceptable early oncological outcome with this technique.

In our patients, utilization of the ePRP technique (Inoue et al. 2010) provided adequate control of the DVC and contributed to shorten the operative time. The median operation duration was 196 minutes and it tended to follow a learning curve, as shown in Figure 4. We believe that it is possible to reduce the operative time when 2 surgeons work in concert. In contrast, estimated blood loss did not follow a learning curve. Estimated blood loss exceeded 2000 ml in 6 cases (37.5%), whereas bleeding from the DVC was effectively controlled in all cases. We think that prostate size and/or tumor stage might be correlated with intraoperative bleeding volume.

Neurapraxia and rhabdomyolysis are rare but major complications related to an exaggerated lithotomy position, and were noted in 1 of our cases. Older age, inappropriate lithotomy position, and especially prolonged operation time are thought to be the main risk factors for developing these complications (Anema et al. 2000; Gumus et al. 2002; Price et al. 1998; Hindley & Watson 2007). Our patient (case 15) seem to develop neurapraxia and rhabdomyolysis in direct proportion to the duration of the exaggerated positioning. Thus, these complications might be reduced to a minimum along with improvements in our surgical technique to provide a shorter operation time.

The recent trend of surgical procedures is to seek a minimal invasive potential, such as laparoscopic or robotic-assisted radical cystectomy, rather than an open approach. Several studies have demonstrated that a robotic assisted radical cystectomy for locally advanced bladder cancer patients was technically feasible with acceptable complication rates and early oncological outcomes (Hayn et al. 2010; Kauffman et al. 2010; Hosseini et al. 2011). However, an open approach may be advocated in cases in which the urethra must be included with the excised specimen, and/or with prior pelvic medical or surgical treatment. We believe that the present hybrid procedure using perineal and abdominal approaches will ultimately contribute to improvements in the prognosis of such affected patients.

A major limitation of the present study is the small number of cases studied. In addition, our median (range) follow-up period for alive patients was only 20 months. Some observations have suggested that the median time to any cancer recurrence after a radical cystectomy is about 1 year and that nearly 90% of recurrences occur within 3–4 years (Mitra et al. 2011; Stein et al. 2001; Solsona et al. 2003). Hence, data from longer follow-up periods are needed before definitive conclusions can be made regarding the oncological efficacy of a prostatocystectomy using our hybrid procedure.

## Conclusion

The present prostatocystectomy performed under a hybrid procedure using perineal and abdominal approaches is a well-organized method that can provide good visualization of the surgical structure around the prostate, leading to a reduction in or prevention of local recurrence, as well as surgical complications such as rectal injury, even in patients who underwent prior pelvic irradiation or pelvic surgical treatment. Our initial experience is encouraging, though additional study and more definitive long-term data are necessary.
